# Sex and Gender Bias in Kidney Transplantation: 3D Bioprinting as a Challenge to Personalized Medicine

**DOI:** 10.1089/whr.2020.0047

**Published:** 2020-07-20

**Authors:** Manon van Daal, Maaike E. Muntinga, Sandra Steffens, Annemie Halsema, Petra Verdonk

**Affiliations:** ^1^ Department Medical Humanities, Amsterdam Public Health Research Institute, Amsterdam UMC-VUmc, Amsterdam, The Netherlands.; ^2^ Department of Curriculum Development, Hannover Medical School, Hannover, Germany.; ^3^ Faculty of Humanities/Philosophy, Amsterdam VU, Amsterdam, The Netherlands.

**Keywords:** 3D bioprinting, kidney, gender, sex, transplantation

## Abstract

In this article, we explore to what extent sex and gender differences may be reproduced in the 3D bioprinting of kidneys. Sex and gender differences have been observed in kidney function, anatomy, and physiology, and play a role in kidney donation and transplantation through differences in kidney size (sex aspect) and altruism (gender aspect). As a form of personalized medicine, 3D bioprinting might be expected to eliminate sex and gender bias. On the basis of an analysis of recent literature, we conclude that personalized techniques such as 3D bioprinting of kidneys alone do not mean that sex and gender bias does not happen. Therefore, sex and gender considerations should be included into every step of developing and using 3D-bioprinted kidneys: in the choice of design, cells, biomaterials, and X-chromosome-activated cells.

## Introduction

Personalized medicine (PM) envisions “personalized health care solutions” with the aim to achieve optimal patient outcomes, the highest safety margin, and the lowest possible costs.^1–4^ Core tenet of PM is the consideration of individuals' unique genetic makeup in interaction with the external environment, which enables timely diagnoses, tailored risk assessments, and optimal treatments.^2^ It has been suggested, however, that current practices insufficiently account for aspects of human diversity, in particular sex and gender.^1,3^ Without concerted effort, PM approaches might reproduce sex and gender bias.^5^

In this article, we explore whether PM-based technologies, such as 3D bioprinting, can help eliminate sex and gender bias. Gender bias occurs when sex and gender are insufficiently considered in research and clinical practice.^6,7^ A consequence is suboptimal care for both women and men. Not accounting for sex/gender throughout the research process could lead to inaccuracies, research inefficiency, and sustain existing biases or even manifest novel biases, which in turn could uphold health inequities.^8^

Furthermore, we elaborate on how sex and gender bias might be reproduced by emerging technologies, in particular 3D-bioprinted kidneys, a technique that uses individual cells to create exact copies of organs for transplantation purposes. Our focus on kidney transplantation is informed by urgency: kidneys are by far the most transplanted organ globally.^9^ Access to 3D-bioprinted kidneys would mean fewer kidney patients relying on donor programs to find a matching donor, which makes 3D bioprinting likely to be widely implemented once available.

Sex and gender differences impact aspects of kidney care, such as diagnosis, treatment, and organization of care.^10^ If gender bias is reproduced in PM techniques such as 3D bioprinting, the potential broad uptake of 3D bioprinting of kidneys means that a large group of people might be exposed to the negative consequences of sex and gender bias. Conversely, if sex and gender are adequately accounted for, 3D bioprinting might contribute to the elimination of such bias.

In this article, we adhere to the definition of sex and gender as currently used in medical research (*e.g.*, CIHR, gendered innovations), although we do acknowledge that “sex” and “gender” cannot simply be distinguished, and that understanding “sex” as binary might be the result of dominant ideas about gender.^8,11,12^ We differentiate between sex as masculinity and femininity in terms of genes, genitals, and gonads (“sex as 3G-gender”) and gender, that is, behavior and sociocultural influences.^13,14^ We define sex and gender bias as follows: “a systematic error (…) leading to the mistaken view of men and women as similar (or different) in exposure to risks or in the natural history of disease (…).”^15^

In the first section, we describe sex and gender differences in kidney transplantation and donation. In the second section, we explore sex and gender bias in 3D bioprinting of kidneys. We conclude that personalized techniques such as 3D bioprinting alone do not prevent sex and gender bias from occurring. Instead, sex and gender considerations should be included into every step of the 3D bioprinting process.

### Sex and gender differences in kidney donation and transplantation

Sex and gender play a role in clinical processes, treatment opportunities, and outcomes of organ donation and transplantation. For instance, sex and gender influence who donates (the donor) and who receives (the recipient). Living organ donation is prone to gender influences, because it allows people to decide what organ they will donate and to whom.^16^ More women than men donate their organs, which is linked to the gendered role of being a caregiver, and to gender differences in altruism, empathy, and the desire to help.^17–19^

Furthermore, disease-specific sex and gender differences play a role. For instance, men are generally more often transplant recipients as a result of the higher incidence of end-stage diseases among men.^19^ Sex and gender differences occur after organ transplantation as well. An important difference is that female organs, including female kidneys, are more often rejected by all-gender bodies because of immune response-related processes.^16,20,21^ Differences in alloantibody levels, such as the antihuman leukocyte antigen, can complicate the organ donation and transplantation process.^16^ Women generally have higher (allo)antibody levels because of pregnancies, and also due to X-chromosomes, of which most women have two and most men one, that contain more genes for immunity.^21^

Finally, sex and gender impact transplantation outcomes: men and women respond differently to immunosuppressive drugs, and there are differences in post-transplantation infection rates.^22^ Clinical studies show that the weight-normalized oral bioavailability, the drug dosage that finally reaches the therapeutic site of action, of immunosuppressive drugs such as cyclosporine is significantly higher in women than in men. This means that women on average need a higher dosage of cyclosporine than men.

Furthermore, infections, including HIV, BK virus, and tuberculosis, are very common after organ transplantation and can ultimately affect graft survival. A study showed that male sex is a risk factor for BK virus infection.^22^ BK virus is a fairly harmless virus, which the majority of people receive during childhood. However, as soon as the immune system is suppressed, such as after a kidney transplant, this virus can reappear and enter the kidney, eventually causing rejection of the organ.^22^

There is increasing evidence of the impact of sex and gender on kidney donation and transplantation. First, there is an imbalance among living kidney donors: in 2016, female donors in The Netherlands outnumbered female recipients, while the opposite was true for male donors.^23^ Research suggests physicians consider their male kidney patients more often eligible for transplantation, whereas female patients less often receive dialysis; it has been suggested that the latter is related to physicians' assumption that female patients are frailer than they actually are.^20,24^

Moreover, differences in kidney volume cause female donor kidneys to be more often rejected after transplantation than male donor kidneys.^19^ Kidney volume is positively associated with successful post-transplant graft outcomes.^25,26^ Smaller kidneys have a reduced nephron number, which is associated with poorer kidney survival.^16,19^ Although larger persons can receive smaller organs, smaller persons cannot receive larger organs.^20^ Male donors generally have larger kidneys, and therefore, female patients face more difficulties finding a donor match.

Sex and gender also influence post-transplant mortality. Studies show male recipients have lower 5-year survival rates after transplantation than female recipients.^19^ No clear explanation yet exists, but it is hypothesized female estrogens are a protective factor in long-term graft outcomes.^19,22^ Mortality differences might also be attributed to lifestyle factors: male patients are more likely to take poorer care of their graft, to continue unfavorable habits, or to miss follow-up visits.^19^ The above suggests that, if unaccounted for, sex and gender differences could cause unequitable outcomes of kidney transplantation.

### 3D bioprinting: the end of gender bias?

3D bioprinting, an emerging biotechnological innovation, might offer a solution to prevent and reduce disparities. “3D” refers to the three-dimensional printing of biomaterials, for example, blood vessels, bones, and vascular networks with human cells.^27,28^

Recent evaluations suggest 3D bioprinting could transform clinical responses to a range of conditions and diseases, such as organ failure.^28^ The technique makes use of multiple print heads to press out different cell types, together with polymers, that help to keep printed structures in shape.^27^ Bio ink, a biomaterial that conveys living cells through a printing process, is used to design these 3D structures.^28–30^ So-called scaffolds of biomaterial help grow these cells into tissues. The 3D bioprinters currently tested can create functional human tissue, such as kidney and liver tissue.^31^

The field of 3D bioprinting is rapidly evolving. Researchers have been building and testing accurate models of human organs to better understand the molecular mechanisms of disease development, or the effect of drugs on nephrotoxicity.^31–33^

When 3D-bioprinted tissues become available for transplantation in clinical settings, they will be customized to fit the unique (genetic) profile of a single patient.^28^ One might argue this once and for all ends sex and gender bias in kidney donation and transplantation. Unfortunately, such an effect is highly unlikely if we do not uphold structural and ongoing attention for sex and gender differences in research and practice. We illustrate our point by discussing sex and gender factors that emerge across different stages of the 3D bioprinting decision-making process: choice of design, choice of cells and materials, and choice of X-chromosome-activated cells.

First, sex and gender issues manifest in the 3D bioprinting model choice. The main goal of 3D bioprinting is to restore the patient's original health and improve quality of life. Bioengineers can choose between *in vivo* and *ex vivo* applications of the 3D-bioprinted construct, and both applications require the inclusion of sex and gender differences to avoid gender bias.

When bioengineers use *in vivo* applications, they mimic the targeted organ or tissue to transplant back into the patient's body. *In vivo* therefore requires certain design requirements such as shape, size, and resolution.^34^ The design of a 3D-bioprinted organ starts with bioengineers using CT scans of the original organ or tissue to print anatomically accurate constructs.^34^ The 3D-bioprinted organ will maintain structural, mechanical, biological, and metabolic properties similar to those of a normal and healthy organ.^35^

The final construct is either a direct copy of the patient's own organ, or it is computer generated. It has been suggested that in the computer-based model, sex and gender issues related to acceptance or rejection of the organ by the body might persist, whereas in the direct copy, such issues disappear.^35^ Computer-generated models are based on blueprints of existing organs. Such organs can be derived from a male or female source. Because sex differences in organ anatomy influence graft survival,^25,26^ computer-generated models might reproduce gender bias and thus unequal transplantation outcomes.

Conversely, direct copies use the receiver's own organ as a blueprint and are therefore expected to reduce potential problems around organ rejection, including sex and gender issues.^29,35^ However, other issues persist. A final printed product might not be similar to the blueprint. Kerestes^35^ showed that in the 3D-bioprinted organ, cells are more tightly grouped together, which causes shrinking of the final 3D structure compared with the original.^35^ Since women already have smaller kidneys than men, a further shrinking process may result in poorer graft outcomes, which must be taken into account.

As mentioned before, another functional application of 3D-bioprinted organs could be their use outside the body (*ex vivo*), for example, permanently or temporarily attached to the body comparable with an ostomy or a dialysis machine. Such *ex vivo* applications are solely aimed at restoring functionality and therefore do not have to mimic the *in vivo* organs or tissue. However, such designs might raise new questions about potential gender and sex influences.

Literature about the experiences of colostomy and ostomy users, for example, points at sex and gender differences regarding body image and sexual functioning; in general, female patients experience more adverse effects than males.^36^ Current *ex vivo* solutions for renal patients (peritoneal dialysis [PD] and hemodialysis) show distinct sex and gender differences in patient profiles, user experiences, and treatment outcomes.^37–39^ Although men have higher prevalence rates of chronic kidney disease with and without receiving hemodialysis,^38^ male survival rates fall below female survival rates upon receiving PD,^37^ women might be less likely to opt for an arteriovenous (AV) shunt, a technique that enhances health outcomes.^39^ So far, these differences remain poorly understood. Moreover, questions arise about how gendered determinants of acceptability and patient satisfaction such as weight, beauty norms, and work will affect people with *ex vivo* application.

Second, sex and gender issues manifest in the choice of cells and materials. Preferred cells for tissue-engineering are pluripotent stem cells (PSCs), because of their ability to self-renew and differentiate into any required adult cell type.^29,30^ PSCs are divided into embryonic stem cells (ESCs) and induced PSCs (iPSCs).^29^ The use of both ESCs and nonautologous iPSCs requires a stem cell donor (*e.g.*, a family member), which warrants attention for sex and gender differences between donor and recipient—for instance, the abovementioned gender imbalance among donors and the differences in transplantation outcomes. Autologous iPSCs can be transformed into a state that is similar to ESCs and could possibly replace them, but do require the consideration of additional factors.

So far, insight in transplantation risks, including transplantation of 3D-bioprinted iPSCs, is lacking because iPSCs have not been inserted into a human body in this way before.^28,30,40^ However, using autologous iPSCs could overcome sex and gender differences in transplantation outcomes for several reasons. For one, such cells do not normally induce a toxic or immune response because they are not foreign, and therefore, the risk of graft rejection is minimized.^29,35^ In addition, the risk of infection decreases due to a low level of transmittable disease risk.^35^ Women have stronger immune systems and tend to reject donor organs sooner, and could potentially benefit from the use of autologous iPSCs. Conversely, women's stronger immune systems could also cause higher rejection rates of printed organs.

Knowledge gaps in this area warrant further research into the effects of sex and gender on 3D bioprinting with stem cells. For instance, it is uncertain whether ESC- and iPSC-based donor prints will be identical to patients' own cells, or whether and how male and female PSCs differ. Evidence shows muscle-derived stem cells of female mice generate new muscle tissue much faster than male mice stem cells when transplanted into diseased muscle of mice of either sex.^41^ This effect might be present in other stem cells as well, including human ones.^41^

Furthermore, after cell type selection, a next step is the selection of substance in which to suspend the cells or scaffold material to support bioprinting of 3D compositions, such as natural polymers or synthetic hydrogels.^29,42^ So far, effects of these materials on stem cell properties—and thus on the manifestation on sex differences—are largely unknown. If sex differences in stem cell properties such as tissue regeneration capacity prove to be clinically relevant, they could determine medical guidelines and standards of care.

Third, sex and gender issues might play a role in the choice for X-chromosome-activated cells in 3D bioprinting. More specifically, the presence of X- or Y-chromosomes might have consequences for transplantation outcomes, although so far the particularities of this relationship are unknown. For example, sex could potentially be significant because X-chromosomes carry a high number of genes for immunity, which could increase the likelihood of rejection of the 3D-bioprinted organ.

In addition, when 3D bioprinting a female organ, bioengineers decide which X-chromosome-activated cells to use. However, in women, the activation of X-chromosomes is not a synchronized process: early in the development of the female embryo, one of the two X-chromosomes is randomly inactivated in each cell, with the exception of the reproductive cells.^43^ Activation and inactivation happen randomly and continuously, resulting in an “epigenetic mosaic”: a construction of cells in which both X-chromosomes are alternately activated. Because X-chromosome activation is triggered by environmental, epigenetic, and genetic factors, both sex and gender factors might play a role in this process.

As a result of X-chromosome inactivation, a female 3D-bioprinted organ will never be an exact copy of its original; in theory, a copied and printed organ could even contain the exact opposite activated X-chromosomes than the original organ. More complex even, printed organs are products of one single cell, while the original organ is a mosaic, a variation between the two X-chromosomes. For female transplant patients, this means that although their 3D-printed kidney graft might be designed with autologous cells, it will never be an exact copy of the original kidney. This discrepancy could influence the transplantation outcomes and how the organ functions, eventually introducing structural differences in the clinical trajectories of male and female patients.

Efforts to print personalized and high-functioning kidneys for male and female transplant patients are further complicated by new and old discoveries in the field of sex-chromosome mosaics (small groups of genetically distinct cells) and chimerism (massive input of genetically distinct cells). DNA sequencing has shown that human tissue can consist of a patchwork of genetically distinct cells of various sexes (*e.g.*, both XX- or XY-chromosomes), which do not match the phenotypic sex of the individual without causing challenges for health.^12,44^

There is one form of such chimerism that is widespread, namely microchimerism (Mc). This occurs when stem cells from a fetus cross the placenta into the mother's body and vice versa^12,45^ and persist long term in both.^45,46^ In theory, these crossover cells are foreign to the body and should be rejected. These microchimeric cells penetrate to every cell and tissue in the body, and have been identified in bone marrow-derived stem cells, liver, gallbladder, intestine, heart, kidneys, and even brains.^12,45^

Microchimeric cells are mostly found in XY men (carrying cells of their mother) and mothers who gave birth to XY sons. However, sometimes, microchimeric cells have been found in women with no history of pregnancy, which indicates the existence of other potential sources of Mc, for instance, miscarriage, twin births or older siblings, or history of blood transfusion.^45^ The existence of microchimeric cells means that organs and tissues can contain female cells when the majority of cells are male, and vice versa. If a 3D-bioprinted construct does not account for microchimeric cells (a likely scenario), this might have consequences. So far, evidence of how the presence of XX cells in an XY male, or the reverse, affects tissue or organ characteristics and eventually health is only slowly emerging.^12^

Gammill and Nelson^45^ described the sex-related consequences of Mc, such as the occurrence of systemic sclerosis in women and the adverse effect of Mc on graft-versus-host-disease outcomes in women. No information exists as to how Mc affects function and success of 3D-bioprinted constructs. Overall, inactivation of X-chromosomes and Mc might complicate the personalization and optimization of 3D-bioprinted constructs, and challenges 3D bioprinting's potential to contribute to the elimination of sex and gender bias.

## Conclusion

We pointed out how sex and gender issues might manifest in different stages of the 3D bioprinting process of human kidneys. The particularities and complexities of these manifestations have so far remained unexplored. We argue that although 3D bioprinting could be a step forward in the elimination of sex and gender bias in kidney transplantation by reducing donor dependency, 3D bioprinting could also reproduce such bias, or even introduce new biases.

Paradoxically, 3D bioprinting might inadvertently contribute to disparities in transplantation outcomes of male and female patients. To allow 3D bioprinting to fulfill its potential as a more personalized solution in kidney donation and transplantation, it is therefore essential to continuously consider sex and gender when developing, testing, and implementing new approaches.

Finally, mechanisms that reproduce sex and gender bias in innovative PM technologies might not be limited to the field of organ transplantation. It is therefore essential that bioengineers, researchers, and clinicians gain more insight in the potential impact of sex and gender on molecular and cellular properties, *in vivo* and *ex vivo* processes, and eventually treatment outcomes of PM-based interventions. As always, more research is needed if we want PM to equally benefit future patients of all genders.

## Authors' Contributions

M.D. conceptualized the article and invited M.M., A.H, and PV. to collaborate. M., M.E., A., and P. structured and drafted the article. S.S., a urologist, was later invited to give her medical opinion about the article and (re)wrote the section about sex and gender differences in kidneys. All authors contributed to the intellectual content and authorship. All authors commented on and revised the article.

## Abbreviations Used

AV: arteriovenous
CKD: chronic kidney disease
ESCs: embryonic stem cells
GVHD: graft-versus-host-disease
HLA: human leukocyte antigen
iPSCs: induced pluripotent stem cells
Mc: microchimerism
PD: peritoneal dialysis
PM: personalized medicine
